# Risks and benefits of animal-assisted interventions for critically ill patients admitted to intensive care units

**DOI:** 10.1186/s44158-023-00100-y

**Published:** 2023-05-31

**Authors:** Marco Fiore, Andrea Cortegiani, Giansaverio Friolo, Francesca Frigieri Covani, Luigi Cardia, Fausto Ferraro, Daniela Alampi

**Affiliations:** 1 Department of Women, Child, and General and Specialized Surgery, University of Campania Luigi Vanvitelli, Naples, Italy; 2 Department of Surgical, Oncological and Oral Science (Di.Chir.On.S.), University of Palermo, Palermo, Italy; 3 Department of Anesthesia, Intensive Care and Emergency, University Hospital Policlinico Paolo Giaccone, Palermo, Italy; 4 Sham Italia – Relyens group, Milan, Italy; 5 Department of Intensive Care, Santa Maria Annunziata Hospital, Florence, Italy; 6 Department of Human Pathology of Adult and Childhood “Gaetano Barresi”, University of Messina, Messina, Italy; 7 Department of Clinical and Surgical Translational Medicine, Sapienza University, Rome, Italy; 8 Unit of Anesthesia, Intensive Care and Pain Medicine, Sant’Andrea Hospital, Rome, Italy

**Keywords:** Animal-assisted interventions, Animal-assisted activities, Pet therapy, Animal-assisted therapy, Critically ill patient, Intensive care, Patient-centered outcomes, Systematic review

## Abstract

**Background:**

Pets offer significant health benefits, from decreased cardiovascular risks to anxiety and post-traumatic stress improvements. Animal-assisted interventions (AAI) are not frequently practiced in the intensive care unit (ICU) for fear of health risk for critical patients because there is a hypothetical risk of zoonoses.

**Objectives:**

This systematic review aimed to collect and summarize available evidence about AAI in the ICU. The Review questions were “Do AAI improve the clinical outcome of Critically Ill Patients admitted to ICUs?” and “Are the zoonotic infections the cause of negative prognosis?”.

**Methods:**

The following databases were searched on 5 January 2023: Cochrane Central Register of Controlled Trials (CENTRAL), EMBASE, and PubMed. All controlled studies (randomized controlled, quasi-experimental, and observational studies) were included. The systematic review protocol has been registered on the International Prospective Register of Systematic Review (CRD42022344539).

**Results:**

A total of 1302 papers were retrieved, 1262 after the duplicate remotion. Of these, only 34 were assessed for eligibility and only 6 were included in the qualitative synthesis. In all the studies included the dog was the animal used for the AAI with a total of 118 cases and 128 controls. Studies have high variability, and no one has used increased survival or zoonotic risk as outcomes.

**Conclusions:**

The evidence on the effectiveness of AAIs in ICU settings is scarce and no data are available on their safety. AAIs use in the ICU must be considered experimental and follow the related regulation until further data will be available. Given the potential positive impact on patient-centered outcomes, a research effort for high-quality studies seems to be justified.

**Supplementary Information:**

The online version contains supplementary material available at 10.1186/s44158-023-00100-y.

## Background

The animal-assisted intervention (AAI) definition by The International Association of Human-Animal Interaction Organizations (IAHAIO) is “a goal-oriented and structured intervention that intentionally includes or incorporates animals in health, education, and human services for therapeutic gains in humans.” Animal-assisted activity (AAA), animal-assisted education (AAE), and animal-assisted therapy (AAT) are the different types of AAI; all AAIs involve skilled human/animal teams with an active certification [1]. Therefore, the AAT in a hospital setting differs from the patient visit of their domestic pet.

Several studies focused on AAT in different settings and the most frequently reported measure of effectiveness is depression reduction in patients with dementia [2]. In hospitalized pediatric patients, AAT seems to decrease blood pressure (BP) and control pain [3], although in these signs of efficacy, safety is doubtful as there is a potential risk of zoonosis posed by the involvement of dogs in AAI in healthcare facilities [4]. Otherwise, the benefits seem to outweigh the risks [5].

The role of AAI in intensive care units (ICU) has not been well established, maybe for the critical illness associated with ICU admission and the relative concerns for patient safety.

### Purpose of review

The authors, members of the clinical risk study group of the Italian Society of Anesthesia, Analgesia, Resuscitation and Intensive Care (SIAARTI) have promoted a systematic review of AAI in ICU to summarize the available evidence on this topic. The Review questions were:

- “Do AAI improve the clinical outcome of Critically Ill Patients admitted to ICUs?”
- “Are the zoonotic infections the cause of negative prognosis?”

## Methodology

### Protocol and registration

The systematic review protocol registration in PROSPERO took place on 21 August 2022 (No. CRD42022344539). The registration was performed after the primary databases (the JBI Database of Systematic Reviews, the Cochrane Database of Systematic Reviews, and PROSPERO) search to exclude existing protocols. The systematic review was conducted following the Preferred Reporting Items for Systematic reviews and Meta-Analyses (PRISMA) methodology [6].

### Study search

The literature search was performed from inception to 5 January 2023 using the Population, Intervention, Comparison, Outcome, and Study (PICOS) design methodology (Table 1). The search was performed on Cochrane Central Register of Controlled Trials (CENTRAL), PubMed, and EMBASE. The search strategies for all database searches are in Appendix 1.

**Table 1:** PICOS methodology for the search strategy

Participants	Intervention	Comparison	Outcomes	Study design
Critically ill people admitted to ICUs	Animal-assisted interventions	Usual treatment	Primary: all-cause mortality Secondary: healthcare-associated infections; clinical improvement	RCT, quasi-experimental, observational studies

### Study selection

**Fig. 1:**
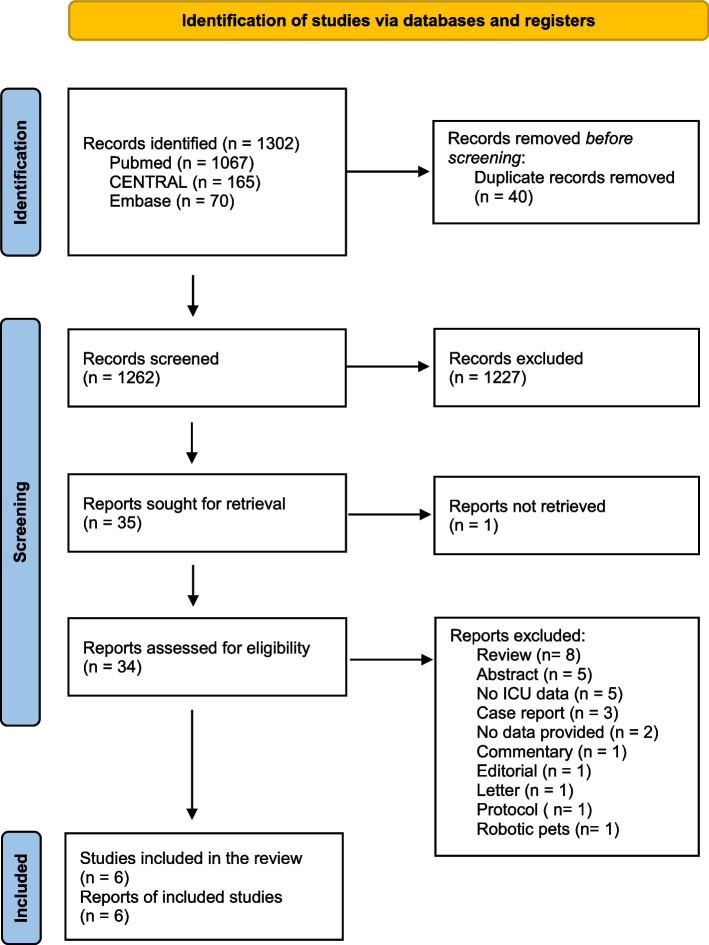
PRISMA flow diagram. CENTRAL, Cochrane Central Register of Controlled Trials

After the search of all databases, the duplicated papers were removed using Endnote software (Endnote VX9, Clarivate Analytics, Philadelphia, PA, USA). Eligible papers were all controlled studies, including randomized clinical trials (RCT) and non-randomized studies (quasi-experimental and observational studies), published in the English language and peer-reviewed. Publication time restriction was not applied. Two authors (DA and MF) screened the title and abstracts of the retrieved papers independently. DA and MF screened the full text of the selected papers for final inclusion. Any eligibility disagreement was resolved by discussion between the above-mentioned authors (DA and MF). The text of the identified studies was assessed in detail independently, recording the exclusion reasons. The results of the identification, screening, and inclusion are presented in a PRISMA flow diagram (Fig. 1).

### Outcomes and definitions

All-cause mortality was the primary outcome. Healthcare-associated infections and clinical improvement were the secondary outcomes. For clinical improvement, we did not adopt a pre-defined description using the outcome established by the author.

### Data extraction and quality assessment

The data were where extracted blindly by two authors (DA, MF). The quality of the included studies was assessed with the Cochrane Data collection form for intervention reviews for RCTs and non-RCTs and the Newcastle–Ottawa assessment scale for case–control and cohort studies.

## Results

A total of 1302 records were identified (PubMed 1067, CENTRAL 165, Embase 70), and no studies were identified via other methods. Forty duplicates of these 1302 records were eliminated before the screening. Thirty-four of the 1262 records screened were assessed for eligibility, of these only six were reported in the final synthesis (Table 2).

**Table 2:** Studies reported in the qualitative synthesis, highlighted in bold the main outcomes reported in the results

Author (Year) [ref.]	Setting	Study design	Timing	Animal experimented (patient number)	Control (patient number)	Outcome(s)	Results for the primary outcome	Interpretation of the finding
Miller (2003) [7]	Open-heart surgery	QES	Hospital discharge	Dog (17)	Usual care (13)	**Discharge teaching**	11/17 dog 10/13 usual care	Not beneficial
Cole (2007) [8]	Advanced heart failure	RCT	Hospital stay	Dog (26)	Volunteer (25); usual care (25)	**PCWP**; Systolic PAP; PCWP; EPI & NE levels; anxiety^a^	Adjusted mean difference (SD), P dog vs volunteer − 3.70 (1.06) .001	Beneficial
Calcaterra (2015) [9]	Pediatric surgery	RCT	Immediate post-surgery	Dog (20)	Usual care (20)	**EEG beta-activity**; DBP; HR; SpO2; HbO2; Pain	20/20 dog 0/20 usual care	Beneficial
Walden (2020) [10]	Pediatric heart transplant	Pre-postQES	Hospital stay	Dog (5)	Dog (5)	**Time (mins),** Distance (ft) walked	14.7 (9.6) no-dog 17.2 (10.3) dog	Beneficial
Branson (2020) [11]	ICU older patients (> 60 y/o)	RCT	Hospital stay	Dog (6)	Usual care (4)	**Stress VAS**; FAS; Salivary CRP, IL-1B, cortisol	Pre Med. Dog 3.5 Post Med. Dog 1 Pre Med. usual 5.5 Post Med. usual 5.5	Beneficial
Jennings (2021) [12]	PICU, CVICU hematology/oncology	RCT	Hospital stay	Dog (44)	Usual care (36)	**Activity level**; Mood; Salivary cortisol	Mean (SD) 5.2275 (13.205) dog 3.889 (11.282) usual care	Beneficial

*CVICU*, Cardiovascular Intensive Care Unit; *DBP*, diastolic blood pressure; *EEG*, electroencephalogram; *EPI*, epinephrine; *FAS*, Faces Anxiety Scale; *ft*, feet; *HbO2*, cerebral prefrontal oxygenation; *HR*, heart rate; *mins*, minutes; *NE*, norepinephrine; *PAP*, pulmonary artery pressure; *PCWP*, pulmonary capillary wedge pressure; *PICU*, Pediatric Intensive Care Unit; *QES*, quasi-experimental study; *RC*, retrospective cohort study; *RCT*, randomized controlled trial; *SpO2*, oxygen saturation; *y/o*, year old; *VAS*, visual analog scale

^a^ Anxiety, sum score in units

The main reason for exclusion was reviews (8 reports), and abstracts (5 reports), of studies with aggregated data not focusing on the ICU setting (5 reports) (Fig. 1).

Of the six studies reported in the synthesis [7–12], three enrolled pediatric patients [9, 10, 12], and three adult patients [7, 11, 12]. Of the studies enrolling adult patients one focused on ICU Older patients (> 60 years old) [11]. The quality of the included studies was low according to the Cochrane risk-of-bias tool (Appendix 2).

## Discussion

In all the studies dog is the animal used in the AAI. No study evaluated mortality as an outcome, even if is the most used outcome measure in the ICU [13]. Indeed, no studies reported on the risk of newly acquired zoonosis, although these infections are the major concern related to AAI [4]. There is a huge heterogeneity in outcomes reported.

Miller et al. in a quasi-experimental study evaluated the dog AAI during discharge teaching, defined as helping patients remember what was taught during hospitalization. In this study, the discharge teaching consisted in watching a video of a duration of ten minutes. The study’s question was “Does the presence of a therapy animal during discharge teaching affect retention of discharge teaching for post–open-heart surgical patients with a median sternotomy incision?”. Thirty patients were enrolled: seventeen in the experimental group (with dog) and thirteen in the control group (usual care). AAI failed to be beneficial during discharge teaching. One possible reason for this outcome was that the experimental group was distracted by AAI during the discharge teaching [7]. Cole et al. in a 3-group RCT evaluated the efficacy of twelve minutes of dog AAI in improving hemodynamic measures and anxiety state in seventy-six patients with advanced heart failure. In one group patients received a 12-min dog AAI; in another group with a 12-min volunteer visit and the third group, patients were treated with the usual therapy. The dog AAI had significantly greater decreases in systolic pulmonary artery pressure and pulmonary capillary wedge pressure compared with both control groups. The dog AAI had significantly greater decreases in endogenous plasma levels of epinephrine ompared to the volunteer dog group. The dog AAI had significantly greater decreases in state anxiety sum score compared with both control groups [8]. Calcaterra et al. in a pilot RCT evaluated the neurological, cardiovascular, and endocrinological impact of dog AAI in response to stress and pain after surgery. The authors enrolled forty children, twenty patients treated with a 20-min dog AAI, and twenty patients treated with the usual therapy. The outcomes explored were electroencephalogram activity, heart rate, blood pressure, oxygen saturation, cerebral prefrontal oxygenation, salivary cortisol levels, and the faces pain scale. The dog AAI had significantly faster electroencephalogram diffuse beta-activity and lower pain perception compared to the control group [9]. Walden et al. in a two-period cross-over study evaluated the ambulation, physiologic stability, patient satisfaction, and perceived benefit of dog AAI in hospitalized heart transplant children. The authors enrolled 5 patients (3 males, and 2 females), the outcomes explored were walking time, blood pressure, and respiratory rate. Therapeutic ambulation was significantly higher in dog AAI (17 min) compared to the control group (15 min) [10]. Branson et al. in an RCT assessed the biobehavioral stress response, anxiety, salivary cortisol, C-reactive protein, and interleukin-1β in older ICU patients treated with 10-min dog AAI compared to usual care. Fifteen patients were enrolled (9 dog AAI, and 6 usual care), and of these, only ten concluded the study. Stress and anxiety were significantly reduced in dog AAI compared to the control group [11]. Jennings et al. in an RCT evaluated the effects of dog AAI on response to stress and activity in pediatric ICU, cardiovascular ICU (CICU), and onco-hematology. Eighty patients were enrolled (44 dog AAI, and 36 usual care). In the experimental group, the patient was treated with a 5–10-min dog AAI, in the control group the patient was treated with the usual therapy. Before and after dog AAI were evaluated cortisol salivary level, activity, and mood of pediatric patients. Dog AAI was significantly associated with decreasing in cortisol salivary level and increasing in mood and activity [12].

Few studies explored the AAI in ICU, no none of these evaluated as an outcome the potential negative impact of bringing animals into the critical care setting. All the studies included in the qualitative synthesis in this systematic review utilized the dog as AAI. Although dogs are the animals that most often cause zoonoses in humans, none of the included studies explored the risk of zoonoses. Infections from dogs to humans can be transmitted through aerosol, stool, urine, and saliva. Bacterial infections transmitted from dogs to humans also include ICU frequent germs such as methicillin-resistant *Staphylococcus aureus* (MRSA) [14].

Recently, Edner et al. explored bacterial transmission from dogs to humans during AAI in a Swedish department of pediatrics at Uppsala University Hospital (UUH). the authors collected samples for cultural examinations before and after the dog AAI: New findings after dog AAI in children were Staphylococcus aureus in the nose-lip area and Pseudomonas aeruginosa in the fingertips, none of the samples for cultural examinations was positive for MRSA, MRSP, VRE, or ESBL-producers [15]. However, the study was conducted in Sweden where the prevalence of these pathogens is negligible. the risk of zoonoses could be disastrous in countries where the percentage of multi-resistant pathogens is high even in communities. Another aspect not evaluated from the few studies exploring AAI in ICU is that dogs in turn can be a vector of infections transmitted from patient to patient.

As regards effectiveness, none of the outcomes studied by the selected studies has any evidence of long-term impact on ICU patients.

The rationale for AAIs in the ICU is remarkable. However, the evidence on the effectiveness of AAIs in ICU settings is scarce and no data are available on their safety, the AAIs deserve to be studied in this setting given the potential positive impact on patient-centered outcomes (PCOs), improvement of the subjective experience during the ICU stay as well as a potential reduction in the incidence of delirium [16].

Given the increasing attention paid to the quality of hospitalization in subjective terms, even in terminally ill patients, with approaches that improve the quality of life of patients and the maintenance of support by their families (including pets) a research effort for high-quality studies is justified for the potential improvement of PCOs.

## Conclusions

The evidence on the effectiveness of AAIs in ICU settings is scarce and no data are available on their safety. AAIs use in the ICU must be considered experimental and follow the related regulation until further data will be available. Future research effort for high-quality studies is justified by a potentially positive impact on PCOs.

## Supplementary Information

**Additional file 1: Appendix 1.** Search strategies.

**Additional file 2: Appendix 2.** **Table S1.** Risk-of-bias assessment in a systematic review of randomized trials, using version 2 of the Cochrane risk-of-bias tool. References (Ref.) are available from the main document. **Table S2.** Risk-of-bias assessment in non-randomized studies of interventions, using ROBINS-I. References (Ref.) are available from the main document.

## Data Availability

The datasets generated during the current study are available from the corresponding author upon reasonable request.

## Abbreviations

CENTRAL: Cochrane Central Register of Controlled Trials
CVICU: Cardiovascular Intensive Care Unit
DBP: Diastolic blood pressure
EEG: Electroencephalogram
EPI: Epinephrine
FAS: Faces Anxiety Scale
HbO2: Cerebral prefrontal oxygenation
HR: Heart rate
NE: Norepinephrine
PAP: Pulmonary artery pressure
PCWP: Pulmonary capillary wedge pressure
PICU: Pediatric Intensive Care Unit
QES: Quasi-experimental study
RC: Retrospective Cohort Study
RCT: Randomized controlled trial
SpO2: Oxygen saturation
y/o: Year old
VAS: Visual analog scale
