# Supplementary material for: Risks and benefits of animal-assisted interventions for critically ill patients admitted to intensive care units
Source: J Anesth Analg Crit Care. 2023 May 31;3:15. doi: 10.1186/s44158-023-00100-y (PMC10245492; doi:10.1186/s44158-023-00100-y)
Supplement: Supplementary file 1 — Additional file 1: Appendix 1. Search strategies. [file 44158_2023_100_MOESM1_ESM.docx]

Search string Embase January 5 2023: 70 results

('intensive care'/exp OR 'care, intensive' OR 'critical care' OR 'intensive care' OR 'intensive care, paediatric' OR 'intensive care, pediatric' OR 'intensive therapy' OR 'paediatric intensive care' OR 'pediatric intensive care' OR 'therapy, intensive' OR 'critically ill patient'/exp OR 'critically ill' OR 'critically ill patient') AND ('animal assisted therapy'/exp OR 'animal assisted therapy' OR 'animal facilitated therapy' OR 'pet therapy'/exp OR 'pet facilitated therapy' OR 'pet therapy' OR 'animal assisted intervention'/exp)

Search string Pubmed January 5 2023: 1067 results

((intensive care) OR (critical care) OR (paediatric intensive care) OR (pediatric intensive care) OR (critically ill patient)) AND ((animal assisted therapy) OR (animal facilitated therapy) OR (pet therapy) OR (pet facilitated therapy) OR (pet therapy) OR (animal assisted intervention))

Search string CENTRAL January 5 2023: 165 results

((intensive care) OR (critical care) OR (paediatric intensive care) OR (pediatric intensive care) OR (critically ill patient)) AND ((animal assisted therapy) OR (animal facilitated therapy) OR (pet therapy) OR (pet facilitated therapy) OR (pet therapy) OR (animal assisted intervention))
