# Supplementary material for: Risks and benefits of animal-assisted interventions for critically ill patients admitted to intensive care units
Source: J Anesth Analg Crit Care. 2023 May 31;3:15. doi: 10.1186/s44158-023-00100-y (PMC10245492; doi:10.1186/s44158-023-00100-y)
Supplement: Supplementary file 2 — Additional file 2: Appendix 2. Table S1. Risk-of-bias assessment in a systematic review of randomized trials, using version 2 of the Cochrane risk-of-bias tool. References (Ref.) are available from the main document. Table S2. Risk-of-bias assessment in non-randomized studies of interventions, using ROBINS-I. References (Ref.) are available from the main document. [file 44158_2023_100_MOESM2_ESM.docx]

**Table S1:** Risk-of-bias assessment in a systematic review of randomized trials, using version 2 of the Cochrane risk-of-bias tool. References (Ref.) are available from the main document.

| **Study** | **Ref.** | **R** | **D** | **Mi** | **Me** | **S** | **O** |
| --- | --- | --- | --- | --- | --- | --- | --- |
| Cole (2007) | [8] | **-** | **+** | **+** | **-** | **-** | **-** |
| Calcaterra (2015) | [9] | **+** | **?** | **+** | **+** | **-** | **+** |
| Branson (2020) | [11] | **+** | **-** | **-** | **?** | **-** | **-** |
| Jennings (2021) | [12] | **-** | **+** | **+** | **+** | **-** | **+** |

**Risk of bias legend**

**R** Bias arising from the randomization process

**D** Bias due to deviations from intended interventions

**Mi** Bias due to missing outcome data

**Me** Bias in measurement of the outcome

**S** Bias in selection of the reported result

**O** Overall risk of bias

**Table S2:** Risk-of-bias assessment in non-randomized studies of interventions, using ROBINS-I. References (Ref.) are available from the main document.

| **Study** | **Ref.** | **D1** | **D2** | **D3** | **D4** | **D5** | **D6** | **O** |
| --- | --- | --- | --- | --- | --- | --- | --- | --- |
| Miller (2003) | [7] | **-** | **-** | **+** | **-** | **+** | **+** | **-** |
| Walden (2020) | [10] | **-** | **-** | **+** | **+** | **?** | **-** | **-** |

**Risk of bias legend**

**D1** Bias due to confounding

**D2** Bias due to selection of participants

**D3** Bias in classification of interventions

**D4** Bias due to deviations from intended interventions

**D5** Bias due to missing data

**D6** Bias in measurement of the outcome

**D6** Bias in selection of the reported result

**O** Overall risk of bias
